# Thymoma Recurrence and its Predisposing Factors in Iranian Population: a Single Center Study

**Published:** 2019-04

**Authors:** Sharareh Seifi, Babak Salimi, Adnan Khosravi, Zahra Esfahani-Monfared, Mihan Pourabdollah, Kambiz Sheikhi

**Affiliations:** 1 Chronic Respiratory Diseases Research Center, National Research Institute of Tuberculosis and Lung Diseases (NRITLD), Shahid Beheshti University of Medical Sciences, Tehran, Iran; 2 Tobacco Prevention and Control Research Center, NRITLD, Shahid Beheshti University of Medical Sciences, Tehran, Iran; 3 Lung Transplantation Research Center, NRITLD, Shahid Beheshti University of Medical Sciences, Tehran, Iran

**Keywords:** Thymoma, Epidemiology, Prognosis, Risk Factors, Recurrence

## Abstract

**Background:**

Thymoma is relatively rare tumor. Prognosis and patients’ outcome vary across different studies. We aimed to study the predisposing factors causing tumor recurrence in thymoma patients.

**Materials and Methods:**

A total of 43 thymoma or thymic carcinoma patients treated at the National Institute of Tuberculosis and Lung Disease (NRITLD), Masih Daneshvari Hospital from September 2005 to January 2017 were evaluated. The primary endpoint was the progression free survival (PFS). The relation of predisposing factors to PFS was studied.

**Results:**

Median age was 55 years old. The mean of follow-up duration was 22.9 months. The most prevalent pathology was thymoma unspecified. Pure red cell aplasia (n=3, 6.9%) was the most prevalent Para neoplastic syndrome. Most of the patients (n=23, 54%) were in stage III and IV Masaoka-Koga staging system. Disease progression was observed in 17 patients (39. 5%). Most recurrences occurred locally. None of demographic characteristics differed between patients who experienced disease recurrence and those who did not. After univariate and multivariate analysis, predisposing factor for disease progression was only Masaoka-Koga stage (P-value=0.015 and 0.031 respectively).

**Conclusion:**

In this study, among different probable predisposing factors, only Masaoka-Koga stage had significant effect on disease recurrence. Large case-control studies may be required for better evaluation of risk factors.

## INTRODUCTION

Primary thymus tumors-thymoma –are rare neoplasms originating from epithelial cells (1). Thymoma is a slow-growing tumor and prognosis is very good if diagnosed in early stages. It is known as the most common mediastinal tumor (2). Annual incidence of thymoma is 1.3–3.2 per 100,000 people per year. (3). Thymoma most frequently reports in the fourth and fifth decades of life and is equally common in men and women. Auto aggressive T-lymphocytes are deleted in thymus medulla and maturation of other T cells develops in thymic epithelial layer (4). Thus, thymus malignancies are commonly associated with abnormality of adaptive immunity and autoimmune disorders such as myasthenia gravis, pure red cell aplasia, or hypogammaglobulinemia (5).

Surgical resection is the primary treatment of thymoma but in unrespectable/inoperable cases, chemotherapy, targeted therapy, and radiation therapy may be considered. Thymoma recurrence is rare and varies according to different studies (6). Some of probable risk factors for recurrence are: disease stage (7), histology (8), incomplete surgical resection of primary tumor (9) and tumor size (10).

As we know, thymoma predisposing factors are relatively unclear and vary from one study to another. Therefore, we aimed to study our institute experiences over 12 years in thymoma patients’ population and assess their outcome and probable disease progression risk factors.

## MATERIALS AND METHODS

Forty three patients with definite thymoma or thymic carcinoma histology who were treated at National Institute of Tuberculosis and Lung Disease (NRITLD), Masih Daneshvari Hospital, were eligible for this cross-sectional, and single institute study from September 2005 to January 2017. This study was conducted according to Shahid Beheshti Medical University’s ethics and scientific local committees (No.: IR.SBM.NRITLD.REC.1396.413) and in compliance with the Helsinki Declaration. Data regarding patient characteristics, stage, tumor size, histology and treatment strategies (surgery, radiotherapy, and/or chemotherapy) were collected. Our institute follows the NCCN and ESMO clinical practice guidelines multi-disciplinary team decision (11, 12). Surgical intervention included extended thymectomy via sternotomy, thoracotomy or video associated thoracoscopy (VATS). Masaoka-Koga staging system (13) was used for disease staging. Histological classification of thymoma was performed according to World Health Organization (WHO) (14) and Suster and Moran grading classification (15). Progression was established by patient’s symptoms, imaging finding and finally pathology proof. Recurrences are classified as local (anterior mediastinum), regional (intrathoracic not contiguous with the thymus), and distant (intrapulmonary and extrathoracic) according to International Thymic Malignancy Interest Group (ITMIG) (16).

### Statistical Methods

The mean ± standard deviation (SD) was calculated for continuous variable. For categorical values number and percentage were obtained. Recurrence or progression of thymoma was considered as main event / endpoint of the study and assessed by progression (or recurrence) free survival (PFS). PFS was defined as the time from diagnosis to documented clinical progression or death for any cause. Patients who were alive or lost at follow up at time of data analysis, were censored for PFS analysis. To compare the frequencies between different groups, Chi-square tests were applied. A P -value of less than 0.05 was considered statistically significant. The predisposing factors including age, gender, smoking status, primary tumor site, histology, treatment strategy, Para neoplastic syndromes and stage in respect to PFS were analyzed using Cox regression tests for univariate and multivariate analysis. All confidence intervals (CIs) for parameters to be estimated were constructed with a significance level of alpha=0.05. Kaplan Meier’s survival curves were obtained for PFS. The log-rank test was used to assess the differences between PFS rates. Never smoker is defined as a person who has smoked less than 100 cigarettes in his/her lifetime (17). IBM SPSS statistical software version 19 for Windows (IBM, Armond, NY, USA) was used for data analysis.

## RESULTS

Samples were obtained by surgical resection in 7 (16.2%), needle biopsy of primary tumors in 27 (62.7%), lung biopsy in 4 (9.3%) and pleural biopsy in 5 (11.6%) patients. Patients’ demographics in relation to recurrence/progression are summarized in Table 1. Median age was 55 years (range 24–83 years). Twenty seven patients (62.8%) were male and 16 (37.2%) were female. About 54% of patients were in stages III and IV. Most common histologic subtypes were unspecified thymoma (n=17, 39.5).

**Table 1.** Demographic characteristics in respect to recurrence.

	**Number(%)**	**Progression**			**P-value**
		Yes	No	NA^a^	
**Age**		N (%)	N (%)		
*<50*	19(44.2)	10(52.6)	2(10.5)	7(36.9)	0.275
*≥51*	24(55.80)	7(29.2)	5(20.8)	12(50)	
**Sex**					
Male	27(62.8)	9(33.3)	6(22.2)	12(44.4)	0.320
Female	16(37.2)	8(50)	1(6.2)	7(43.8)	
**Histology** ^b^					
Thymoma(Unspecified)	17(39.5)	9(52.9)	2(11.7)	6(35.2)	0.831
Thymic carcinoma	10(23.3)	4(40)	2(20)	4(40)	
Thymoma(Type AB)	3(7)	0	1(33.3)	2(66.7)	
Thymoma (type A)	1(2.3)	0	0	1(100)	
Thymoma (type B1)	4(9.3)	1(25)	0	3(75)	
Thymoma (type B2)	4(9.3)	2(50)	0	2(50)	
Thymoma (type B3)	3(7)	1(33.3)	1(33.3)	1(33.3)	
Metaplastic Thymoma	1(2.3)	0	0	1(100)	
**Paraneoplastic syndromes**					
*Yes*	5(11.6)	2(40)	1(20)	2(40)	0.965
*No*	38(88.4)	15(39.5)	6(15.8)	17(44,7)	
**Stage** ^c^					
*I*	17(39.5)	8(47.1)	2(11.7)	7(41.1)	0.778
*II*	3(7)	1(33.3)	1(33.3)	1(33.3)	
*III*	11(25.6)	3(27.2)	3(27.2)	5(45,5)	
*IVa*	9(20.9)	4(44.5)	0	5(45.5)	
*IVb*	3(7)	1(33.3)	1(33.3)	1(33.3)	
**Treatment**					
*Surgery*	3(7)	1(33.3)	1(33.3)	1(33.3)	0.459
*Chemotherapy*	22(51.1)	10(45.4)	2(9.1)	10(45.4)	
*Surgery and adjuvant chemotherapy*	3(7)	0	1(33.3)	2(66.7)	
*Neoadjuvant chemotherapy and surgery*	2(4.7)	1(50)	1(50)	0	
*Chemotherapy and radiotherapy*	7(16.3)	4(57.1)	2(28.5)	1(14.4)	
*Surgery and radiotherapy*	5(11.6)	1(20)	1(20)	3(60)	
*None*	1(2.3)	0	0	1(100)	

Abbreviations:

a: NA: not assessed;

b according to WHO and Suster and Moran classifications;

c according to Masaoka staging system.

Thirty patients (79.06%) received chemotherapy. CAP regimen (cyclophosphamide 500 mg/m^2^, adriamycin 50 mg/m^2^ and cisplatin 50 mg/m^2^ i.v. every 3 weeks) administrated for 29 (88.2%) patients and for rest of them (n=3, 11.8%), paclitaxel(200mg/m2) and carboplatin (AUC 5) i.v., every 3 weeks was used. Chemotherapy -as primary treatment- was administrated over a mean of 4.5 cycles (range 1–6). Details of Para neoplastic syndromes are as follows: 3(6.9%) pure red cell aplasia, one (2.3%) myasthenia gravis and one (2.3%) Good’s syndrome.

### Progression status:

**Figure 1.**
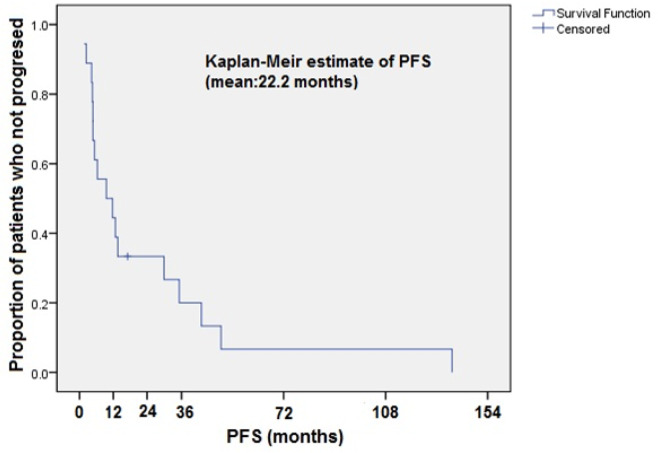
Progression free survival (PFS) in thymoma population. Kaplan-Meier survival curve from onset of recurrence. Mean PFS was 22.02±7.6 months.

**Figure 2.**
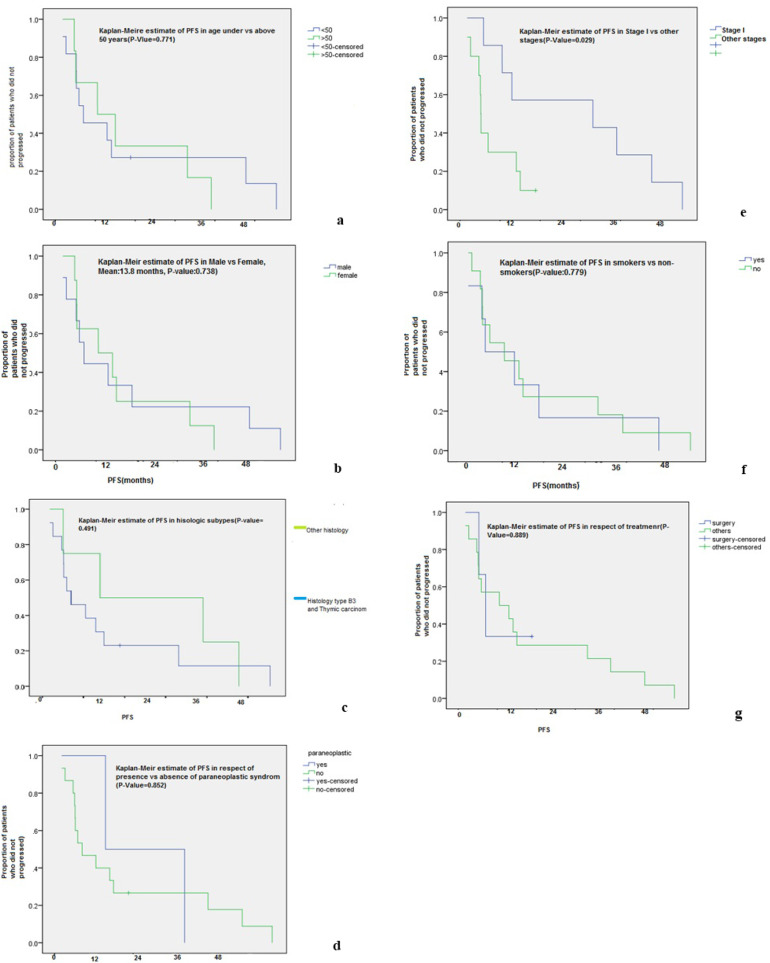
Progression free survival (PFS) in thymoma population respect to different groups of probable recurrence predisposing factors. a: The Kaplan-Meier survival curve from the onset of recurrence for studying the effect of age<50 vs. >50 on PFS; b: The Kaplan-Meier survival curve from the onset of recurrence for studying the effect of Sex on PFS; c: The Kaplan-Meier survival curve from the onset of recurrence for studying the effect of Histology; d: The Kaplan-Meier survival curve from the onset of recurrence for studying the effect of presence vs. absence of Paraneoplastic syndrome; e: The Kaplan-Meier survival curve from the onset of recurrence for studying the effect of Stage on PFS; f: The Kaplan-Meier survival curve from the onset of recurrence for studying the effect of Smoking status on PFS; g: The Kaplan-Meier survival curve from the onset of recurrence for studying the effect Treatment on PFS.

At of the time of data analysis, 9 patients died; among them one death was not associated with thymoma relapse and caused by patient suicide. Information of cases with documented recurrence is shown in Table 3. In 17 patients (39.5%) disease progression was documented. Mean PFS was 15.3±3.6 months. Figure 1 showed Kaplan-Meier survival curve from onset of recurrence. Mean follow up time was 22.9 months. Differences in mean of PFS between different groups of probable recurrence predisposing factors are demonstrated in Figure 2.

**Table 3.** Information of cases with documented recurrence.

**Gender**	**Age (years)**	**Primary treatment**	**Primary Stage**	**PFS (months)**	**Recurrence site**	**Post recurrence treatment**	**Second recurrence**	**Death**
Female	34	Chemotherapy with CAP regimen followed by Radiotherapy	III	10	Distant(Bone and lung)	Chemotherapy with Paclitaxel and carboplatin	No	Yes
Male	48	Chemotherapy with CAP regimen	IVA	2.2	Regional	Chemotherapy with Paclitaxel and carboplatin	Yes	No
Male	47	Chemotherapy with CAP regimen	I	4.9	Local	Patient did suicide before any treatment	No	Yes
Female	68	Chemotherapy with CAP regimen followed by Radiotherapy	I	32.5	Local	Chemotherapy with Paclitaxel and carboplatin	Yes	NA
Female	55	Chemotherapy with CAP regimen	I	27.5	Local	Chemotherapy with Paclitaxel and carboplatin	Yes	No
Male	44	Chemotherapy with CAP regimen	I	10.8	Local	Chemotherapy with Paclitaxel and carboplatin	Yes	Yes
Male	45	Chemotherapy with CAP regimen	IVB	1.5	Distant(Bone and lung(	Chemotherapy with Paclitaxel and carboplatin	Yes	Yes
Female	58	Chemotherapy with CAP regimen	III	3.9	Local	Chemotherapy with Paclitaxel and carboplatin	Yes	Yes
Male	24	Surgery	I	46.2	Local	Re-resection	Yes	Yes
Male	50	Surgery followed by Radiotherapy	II	5.8	Regional	Chemotherapy with CAP regimen	No	NA
Female	63	Chemotherapy with CAP regimen followed by Radiotherapy	IVA	12.5	Local	Chemotherapy with Paclitaxel and carboplatin	No	Yes
Male	57	Chemotherapy with CAP regimen	III	4.2	Regional	Chemotherapy with Paclitaxel and carboplatin	Yes	Yes
Male	47	Chemotherapy with CAP regimen	I	39.8	Local	Radiotherapy	Yes	Yes
Female	42	Chemotherapy with CAP regimen	IVA	4.3	Local	Chemotherapy with Paclitaxel and carboplatin	No	NA
Female	45	Neoadjuvant Chemotherapy with CAP regimen followed by surgery	IVA	4.4	Distant(abdominal lymphadenopathy)	Chemotherapy with Paclitaxel and carboplatin	No	NA
Female	66	Chemotherapy with CAP regimen	I	8.9	Local	Chemotherapy with CAP regimen	No	Yes
Male	83	Surgery	I	121	Distant(Bone and lung)	Chemotherapy with CAP regimen	No	No

Differences in mean of PFS between different groups of probable recurrence predisposing factors are demonstrated in Figure 2. There was only statistically significant difference between stage I vs. other stages (P-Value=0.029).

Predisposing factors in association with PFS were assessed by univariate/multivariate Cox regression analysis (Table 2). Only Masaoka-Koga stage of disease was significantly related to PFS in both univariate and multivariate analysis (P values= 0.015 and 0.031, respectively). Log-rank test was done for each probable progression risk factor including age, gender, disease stage, presence of Para neoplastic syndromes, histology, smoking status and treatment strategies (P-values=0.770, 0.575, 0.029, 0.852, 0.118, 0.969 and 0.273, respectively). Stage is the only statistically significant in association with PFS.

**Table 2.** Prognostic factors affecting progression free survival.

	**Univariate analysis**			**Multivariate analysis**		

	95% CI^a^		P-value	95% CI		P-value
	Lower	Upper		Lower	Upper	
**Age**						
<50 vs≥50	0.294	2.478	0.771	0.256	4.581	0.915
**Sex**						
Male vs. Female	0.254	2.141	0.738	0.087	8.955	0.557
**Histology ^b^**						
Thymoma (Unspecified, Type A, AB, B1 and B2 vs. others)^c^	0.463	4.922	0.494	0.451	9.752	0.345
**Praraneoplasic syndromes**						
Yes vs. No	0. 190	3.941	0.852	0.190	15.914	0.623
**Stage ^d^**						
I vs. others	1.121	2.917	0.015^*^	1.230	69.554	0.031^*^
**Smoking status**						
Smoker vs. non-smoker	0.347	3.010	0.969	0.288	14.20	0.442
**Treatment**						
Surgery vs. other treatment	0.195	4.123	0.889	0.025	1.632	0.134

Abbreviations:

a CI: confidence interval;

b: according to WHO and Suster classifications ;

c: other included type B3 and thymic carcinoma;

d: according to Masaoka staging system.

* significant P- value

### Post recurrence strategy:

As ITMIG classification (16), local recurrence was seen in 10 (58.8%), regional recurrence in 3(17.6%) and distant progression in 4(23.5%) patients. Among them, 13(76.4%) patients were treated with salvage chemotherapy, one patient (5.8%) underwent re-resection of tumor, 2(11.6%) received no further treatment for inappropriate performance status and one patient committed suicide after disease recurrence. Among patients who relapsed, 11 cases showed second progression and there were no differences between Post recurrence strategies and PFS after second recurrence (P-value=0.686).

## DISCUSSION

As far as we know, our study is the first investigation focusing on thymoma progression risk factors in Iranian population. For best appropriate therapeutic approach, we need to identify tumor recurrence predisposing factors, especially in rare tumors.

Thymoma is a slow-growing tumor that has indolent behavior (18). Therefore, the death of the patients has reasons other than thymoma (19). We chose PFS rather than overall survival, as recurrence may state clinical outcomes more accurately than survival. In current study, disease stage was the most important predisposing factor for tumor recurrence that had statistically significant association with PFS in both univariate and multivariate analysis.

Median age in thymoma patients was between 49–56 years in different studies (19–24). Our result is in accordance with them. Aydinar et al. (25) and other studies (26, 27) demonstrated less survival for thymoma patients above 50 years old, but our study did not show that (19,21,22,28).

Ahmad *et al* (29) claimed that female patients had worse prognosis and higher recurrence rate in comparison to men, but in most studies and also in current study equal gender association with prognosis in thymoma was observed (9, 28,30).

Multiple classification systems for thymoma have been defined (28), but currently most of clinicians prefer the WHO classification system. We were unable to review pathologic diagnosis due to long investigation period and also inappropriate storage condition. Thus, we used 2 different histological classification systems: WHO and Suster and Moran classifications.

Different geographic distribution of thymoma subtypes across the globe has been observed. For example in Europe subtype B2 is more common than Asia (31). Some investigators believe B2 and B3 subtypes have unfavorable outcome (32, 10, 20,33), while other studies do not accept it (29,31, 34). In this study, histopathology of tumor had no relation with PFS. Interestingly, we had a rare thymoma histology as metaplastic thymoma. Our case underwent surgery (R0) and due to capsular involvement received radiotherapy after surgery. Up to now, no tumor recurrence has been reported for him. This may suggest that metaplastic thymoma has a benign clinical course according to our and other results (35, 36).

Paraneoplastic syndromes are associated with thymoma, but its role in relation to PFS has been debated. Some investigators demonstrated a protective effect on mortality or recurrence (27, 37, 38), although our results did not confirm any relation between recurrence and paraneoplastic syndromes (the same in many other studies) (20,39). It may be explained by small number of patients with paraneoplastic syndromes in our series and further studies with more number of patients are necessary. About 30–50% of thymoma patients showed myasthenia gravis (40) and 15% of myasthenia gravis cases have thymoma (41). In our series, the most common paraneoplastic syndrome was pure red cell aplasia which may be due to lacking of the capacity to propagate the maturation of immature naive CD4 T cells and export mature naive T cells into the periphery.

Masaoka-Koga stage is known as the most important prognostic factor for recurrence in many investigations (19, 20, 21, 23, 37, 38, 39, 42). In concordance with mentioned studies, Masaoka-Koga stage was an important predisposing factor for tumor recurrence in our patients.

Surgical resection is the gold standard treatment for thymoma. According to some studies, incomplete resection was the predisposing factor for disease progression (10) and complete resection is related to better survival and longer PFS (43). This result suggests patients who tolerate surgery, may get better results than other non-surgical treatment. Other treatment plans including radiotherapy, chemotherapy and multidisciplinary approaches are controversial. Radiotherapy commonly has been implicated in adjuvant setting after R0 stage II and III, after R1 resection in any stages and also in neoadjuvant setting (39). For example, some studies observed better PFS with adjuvant RT in some situations (38,39). Chemotherapy in both adjuvant and neoadjuvant settings has been administrated in thymoma especially in advanced stages and unresectable tumors. Lucchi et al. observed better survival with chemotherapy in advanced stages of thymoma (44). Some investigations reported long-term survival improvement after re-resection with recurrence (45), but other studies did not show that (46). In our opinion it seems that reoperation may be recommended for Thymoma relapse whenever complete resection is possible. Similar to other studies (6), most of the recurrences in our series were local.

There are not many articles focusing on association between smoking status and Thymoma outcomes. Similar to our study, another investigation (47) found no relation between smoking habit and Thymoma prognosis.

The risk of secondary malignancies may be increased in Thymoma patients. Some investigators believe that deregulation of immune system and also kind of treatment (especially radiotherapy) have crucial role in inducing secondary malignancies (48). In our series no secondary malignancy was observed.

Currently, some genetic and epigenetic alternations such as epidermal growth factor receptor amplification, HER2/neu over expression (49), and c-Kit (CD 117) (50) activating mutation has been considered in thymoma pathogenesis (28). Due to rarity of thymoma, genetic study is not performed in routine practice and further studies are needed to show their relation with treatment and prognosis.

The most important limitation of our study was the retrospective nature of study and varying classification systems which may cause significant bias.

In conclusion Masaoka-Koga stage is the most important predisposing factor for disease recurrence among other factors. Further studies with larger number of patient’s cohort, uniform classification system and tumor molecular characteristics are needed to identify prognostic and predisposing factors to improve patient’s survival.
